# Circulating primers enhance platelet function and induce resistance to antiplatelet therapy

**DOI:** 10.1111/jth.13022

**Published:** 2015-06-25

**Authors:** T A Blair, S F Moore, I Hers

**Affiliations:** 1 School of Physiology and Pharmacology, University of BristolBristol, UK

**Keywords:** aspirin, drug resistance, epinephrine, insulin-like growth factor-1, P2Y12 purinoceptor antagonists

## Abstract

**Background:**

Aspirin and P2Y_12_ antagonists are antiplatelet compounds that are used clinically in patients with thrombosis. However, some patients are ‘resistant’ to antiplatelet therapy, which increases their risk of developing acute coronary syndromes. These patients often present with an underlying condition that is associated with altered levels of circulating platelet primers and platelet hyperactivity. Platelet primers cannot stimulate platelet activation, but, in combination with physiologic stimuli, significantly enhance platelet function.

**Objectives:**

To explore the role of platelet primers in resistance to antiplatelet therapy, and to evaluate whether phosphoinositide 3-kinase (PI3K) contributes to this process.

**Methods and Results:**

We used platelet aggregation, thromboxane A_2_ production and *ex vivo* thrombus formation as functional readouts of platelet activity. Platelets were treated with the potent P2Y_12_ inhibitor AR-C66096, aspirin, or a combination of both, in the presence or absence of the platelet primers insulin-like growth factor-1 (IGF-1) and thrombopoietin (TPO), or the Gz-coupled receptor ligand epinephrine. We found that platelet primers largely overcame the inhibitory effects of antiplatelet compounds on platelet functional responses. IGF-1-mediated and TPO-mediated, but not epinephrine-mediated, enhancements in the presence of antiplatelet drugs were blocked by the PI3K inhibitors wortmannin and LY294002.

**Conclusions:**

These results demonstrate that platelet primers can contribute to antiplatelet resistance. Furthermore, our data demonstrate that there are PI3K-dependent and PI3K-independent mechanisms driving primer-mediated resistance to antiplatelet therapy.

## Introduction

Platelet hemostasis is a tightly regulated process mediated by various feedback control mechanisms and key signaling receptors. Disruption of these regulatory controls leads to thrombosis, which, in turn, can trigger the development of an occlusive clot and subsequent cardiovascular complications. Typically, patients who present with pathologic thrombosis undergo pharmacologic intervention with antiplatelet compounds to minimize the risk of developing acute coronary syndrome (ACS). The current ‘gold standard’ preventive measure employed by clinics to treat thrombosis involves the administration of antiplatelet drugs that target cyclooxygenase-1 (COX-1) (e.g. acetylsalicylic acid [ASA]; aspirin) and the platelet P2Y_12_ receptor (e.g. clopidogrel, prasugrel, or ticagrelor).

ASA irreversibly inhibits the conversion of arachidonic acid (AA) to thromboxane A_2_ (TxA_2_), an important positive feedback mediator involved in platelet activation. The inhibitory effect of ASA is achieved by acetylation of Ser529 in the active site of COX-1, blocking the enzyme's critical role in AA metabolism 1,2. Clopidogrel and prasugrel are thienopyridines whose active metabolites covalently bind to the platelet P2Y_12_ receptor and irreversibly inhibit ADP-mediated platelet function 3,4. ASA and platelet P2Y_12_ receptor antagonists may be prescribed to patients as monotherapy; however, they are also administered via a dual antiplatelet regimen as a preventive measure against thrombotic vascular events 5,6. Dual therapy remains controversial, as the PCI-CURE and CARESS trials have demonstrated major improvements in the clinical outcomes of patients receiving combined treatment 7,8, whereas other studies have shown no additional benefit of ASA treatment in the presence of strong P2Y_12_ receptor blockade 9.

The current antiplatelet compounds are limited in their efficacy, as various clinical studies have demonstrated a decreased response, or ‘resistance’, in certain patient subgroups 10. There are several reasons why patients may show resistance to ASA, e.g. reduced absorption/increased metabolism of ASA, the presence of TxA_2_-independent platelet activation pathways, and genetic polymorphisms in the genes encoding COX-1 11,12. Both clopidogrel and prasugrel are prodrugs that require conversion by hepatic cytochrome P450 (CYP) enzymes to become active metabolites *in vivo* 13,14. Therefore, poor bioavailability resulting from genetic polymorphisms in CYP may cause clopidogrel/prasugrel resistance 15. Furthermore, poor bioavailability may also be induced by drugs that interfere with hepatic metabolism 16. Other potential mechanisms underpinning clopidogrel resistance include conditions resulting in increased release of ADP, underdosing, and ADP-independent platelet activation pathways 17.

Patients who are resistant to antiplatelet compounds have been found to show a hyperactive platelet phenotype 18–21. It is well documented that platelet hyperactivity can be induced by elevated levels of circulating primers 22–25. Platelet primers do not stimulate platelet activation, but, in combination with physiologic stimuli, significantly enhance platelet function. The peptide hormones insulin-like growth factor-1 (IGF-1) and thrombopoietin (TPO) are known primers that enhance platelet functional responses via a phosphoinositide 3-kinase (PI3K)-dependent mechanism 26–28. In addition, catecholamines such as epinephrine can also act as platelet primers when used at suboptimal concentrations 29. Interestingly, recent pharmacogenomics studies have also linked increased expression of IGF-1 and IGF-1 receptor mRNA with ASA resistance 30. Furthermore, coronary heart disease (CHD) patients have been found to have significantly higher serum concentrations of IGF-1, a feature that is believed to contribute to coronary atherosclerosis 31,32. TPO is another common factor that has been found to be elevated in conditions associated with coronary artery disease, such as unstable angina 33,34, and epinephrine levels are increased in patients presenting with hypertension 35, and ischemic heart disease 36,37. Elevated levels of these circulating primers may therefore contribute to platelet hyperactivity and, consequently, to the resistance to antiplatelet compounds demonstrated in a population of patients with ACS. This is of particular clinical interest, as these patients are at significant risk of experiencing reoccurring major cardiovascular events 38,39.

In this study, we therefore sought to determine the role of circulating primers in resistance to antiplatelet compounds, focusing specifically on primers known to be elevated in ACS patients. We used platelet aggregation and *ex vivo* thrombus formation to assess the effects of the highly selective P2Y_12_ antagonist AR-C66096 (ARC) and ASA on platelet function in the presence or absence of the primers IGF-1 and TPO, and the Gz-coupled receptor ligand epinephrine. Our results demonstrate that: (i) platelet primers can rescue the inhibitory effects induced by P2Y_12_ blockade and ASA treatment; and (ii) PI3K plays a critical role in IGF-1-mediated and TPO-mediated resistance, whereas there are PI3K-independent mechanisms driving epinephrine-mediated resistance.

## Materials and methods

### Materials

The platelet agonists used were: protease-activated receptor 1 (PAR-1)-activating peptide (SFLLRN-NH_2_; Bachem, Bubendorf, Switzerland), crosslinked collagen-related peptide (CRP-XL) from R. Farndale (Department of Biochemistry, University of Cambridge, UK), and fibrillar HORM collagen (type I) derived from equine tendon (Nycomed, Konstanz, Germany). The platelet inhibitors used were: ARC tetrasodium salt (R&D Systems, Abingdon, UK), ASA (Sigma-Aldrich, Poole, UK), and wortmannin (Tocris, Bristol, UK). The platelet primers used were: long-IGF-1 recombinant protein (receptor grade – AM001; Immunological and Biochemical Test Systems, Binzwangen, Germany), epinephrine hydrochloride (Sigma-Aldrich), and recombinant human TPO (R&D Systems). d-phenylalanylprolyl-arginyl chloromethyl ketone (PPACK) was from Calbiochem (Merck Chemicals, Watford, UK), and heparin was from Sigma-Aldrich. The commercial TxA_2_ ELISA kit and 3,3′-dihexyloxacarbocyanine iodide (DiOC_6_) were from Enzo Life Sciences (Exeter, UK). All other reagents were from Sigma (Poole, UK), unless otherwise indicated.

### Isolation and preparation of platelets

Venous blood was obtained from healthy volunteers with approval of the local research ethics committee at the University of Bristol. Donors provided written informed consent, and reported not having taken antiplatelet agents in the 14 days prior to donation. Blood was drawn into 4% trisodium citrate (1 : 9, v/v), and acidified with acidic citrate dextrose (1 : 7, v/v; 120 mm sodium citrate, 110 mm glucose, 80 mm citric acid). Washed platelets were isolated as previously described 40, and pelleted in the presence of 140 nm prostaglandin E_1_ and 0.02 U mL^−1^ apyrase (grade VII). Platelets were resuspended at 4 × 10^8^ mL^−1^ in modified HEPES–Tyrode buffer (145 mm NaCl, 3 mm KCl, 0.5 mm Na_2_HPO_4_, 1 mm MgS0_4_.7H_2_O, 10 mm HEPES, pH 7.2, 0.1% [w/v] d-glucose, and 0.02 U mL^−1^ apyrase), and allowed to rest at 30 °C for 30 min prior to experimentation.

### Platelet aggregation

Platelet aggregation was performed with a Chronolog 490-4D aggregometer (Labmedics, Abingdon-on-Thames, UK) at 37 °C under continuous stirring at 1200 r.p.m. Platelets (2 × 10^8^ mL^−1^) were preincubated for 10 min at 37 °C with vehicle (0.2% dimethylsulfoxide/HEPES–Tyrode buffer) or the pharmacologic inhibitors ARC (1 μm), ASA (30 μm), and ASA/ARC, with or without wortmannin (100 nm) or LY294002 (40 μm). The platelet primers IGF-1 (100 nm), TPO (50 ng mL^−1^) and epinephrine (5 μm) were added 5 min prior to stimulation with the PAR-1 agonist SFLLRN or the glyycoprotein (GP)VI agonist CRP-XL. Changes in light transmission were continuously monitored with aggrolink Version 4 (Chronolog Corporation, Havertown, PA, USA) for 5 min.

### Measurement of TXA_2_ generation

TxA_2_ levels were measured with a commercially available colorimetric ELISA kit (Enzo Life Sciences), as previously described 41. In brief, platelet samples from the aggregation reactions were quenched at 5 min with 200 μm indomethacin and 5 mm EDTA to inhibit further production of TxA_2_. Samples were centrifuged for 4 min at 12 000 × *g*, and the supernatant was removed and stored at − 80 °C for subsequent analysis according to the manufacturer's protocol. Thromboxane B_2_, the stable hydrolysis product of TxA_2_, was used as a readout of TxA_2_ production.

### *Ex vivo* thrombus formation

Thrombus formation under flow conditions was determined as previously described 26,42. In brief, anticoagulated blood drawn into 2 U mL^−1^ heparin and 40 μm PPACK was pretreated with vehicle (HEPES–Tyrode buffer) or ARC (1 μm) and ASA (30 μm) in the presence or absence of wortmannin (100 nm), and prelabeled with 1 μm DiOC_6_ for 10 min. Blood was treated with vehicle control or the platelet primers IGF-1 (5 nm or 100 nm), TPO (50 ng mL^−1^) and epinephrine (20 or 100 nm) for 5 min before perfusion at an arterial shear rate of 1000 s^−1^ for 5 min over collagen-coated coverslips (50 μg mL^−1^) in parallel-plate flow chambers. Phase-contrast and fluorescence images of thrombi were captured with a × 40 water immersion objective on a fluorescence microscope (BX51WI; Olmpus, Southend-on-Sea, UK) and a Rolera-XR digital camera. Chambers were flushed with HEPES–Tyrode buffer to remove non-adherent cells, and fluorescent images were taken from at least 15 random microscopic fields of view. Quantification of surface coverage was performed with imagej (National Institutes of Health, Bethesda, MD, USA).

### Calcium signaling

Ca^2+^ measurements were performed as previously described 43. In brief, changes in intracellular Ca^2+^ concentration were measured by spectrofluorimetry in platelets (5 × 10^7^ mL^−1^) loaded with Fura-2 at 37 °C, with stirring. Fluorescence excitation was performed at 340 nm and 380 nm with a Hitachi F-4500 (Hitachi High-Technologies, Maidenhead, UK).

### Statistical analysis

Data were analyzed with graphpad prism 5 software (GraphPad Software, San Diego, CA, USA). All data are presented as mean ± standard error of the mean of at least three independent experiments. Data used in statistical analysis were tested with either a one-way or a two-way anova with a Bonferroni or Dunnett multiple comparison *post hoc* test.

## Results

### Role of P2Y_12_ and TxA_2_ in platelet aggregation mediated by PAR-1 and GPVI

**Figure 1:**
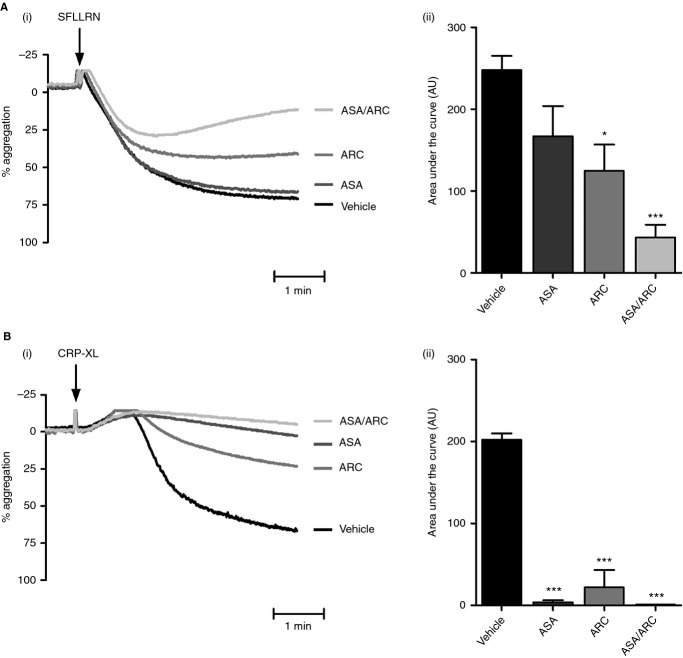
Role of P2Y_12_ and thromboxane A_2_ in protease-activated receptor 1-mediated and glycoprotein VI-mediated platelet aggregation. (A,B) Washed platelets (2 × 10^8^ mL^−1^) were pretreated with vehicle control (HEPES–Tyrode buffer) or inhibitors, i.e. 1 μm AR-C66096 (ARC), 30 μm acetylsalicylic acid (ASA), or ASA/ARC, for 10 min. Platelets were subsequently stimulated with 2 μm SFLLRN (A) or 1.5 μg mL^−1^ crosslinked collagen-related peptide (CRP-XL) (B), and platelet aggregation was recorded for a total of 5 min. Representative aggregation traces (Ai, Bi) and quantified percentage aggregation values (Aii, Bii) are shown. Data are mean ± standard error of the mean, *n* = 3–6. Statistical analysis: one-way anova was used in conjunction with Dunnett's multiple comparison test; *P < 0.05 and ***P < 0.001 as compared with vehicle control.

To examine the role of P2Y_12_ and TxA_2_ in PAR-1-mediated and GPVI-mediated platelet aggregation, platelets were treated with ASA (TxA_2_ inhibitor), ARC (P2Y_12_/ADP inhibitor), or ASA/ARC. ASA (30 μm) had no significant effect on SFLLRN-mediated aggregation, but significantly reduced CRP-XL-mediated platelet aggregation, from 79.7% ± 1.3% to 4.0% ± 3.0% (Fig.1A,B). In agreement with previous studies, P2Y_12_ inhibition with ARC (1 μm) caused a significant reduction in SFLLRN-mediated aggregation 44, with the amplitude being reduced from 75.0% ± 3.6% to 38.0% ± 7.4% (Fig.1A). Similarly, CRP-XL-induced aggregation was drastically reduced from 79.7% ± 1.3% to 12.0% ± 8.5% in the presence of ARC (Fig.1B). Interestingly, combination treatment with ASA and ARC made SFLLRN-mediated aggregation reversible (Fig.1A), demonstrating the importance of TxA_2_ and ADP signaling in sustained platelet aggregation. ASA and ARC completely blocked CRP-XL-mediated aggregation (Fig.1B).

### IGF-1, TPO and epinephrine rescue PAR-1-mediated platelet function in the presence of antiplatelet compounds

**Figure 2:**
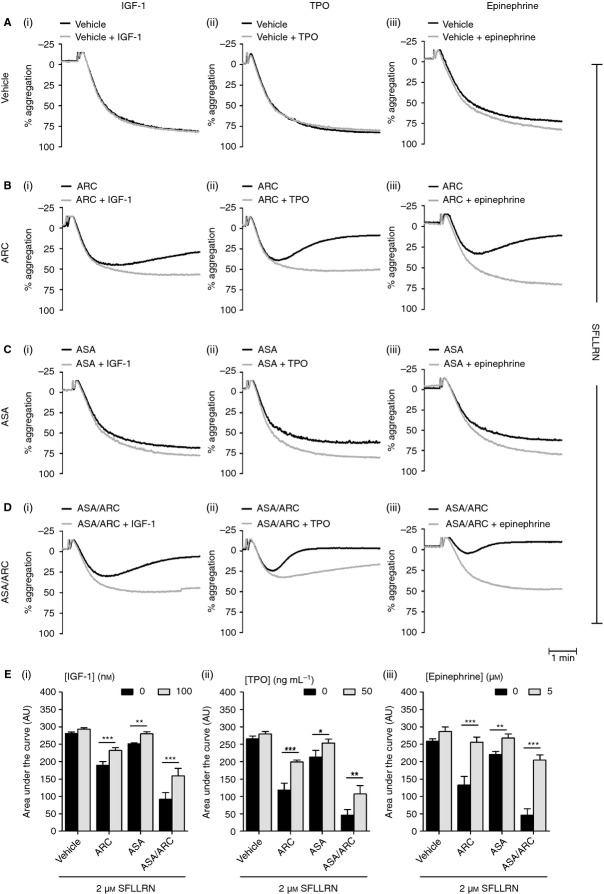
Insulin-like growth factor-1 (IGF-1), thrombopoietin (TPO) and epinephrine rescue protease-activated receptor 1-mediated platelet function in the presence of antiplatelet compounds. Washed platelets (2 × 10^8^ mL^−1^) were pretreated with vehicle control (HEPES–Tyrode buffer) (A) or inhibitors, i.e. 1 μm AR-C66096 (ARC) (B), 30 μm acetylsalicylic acid (ASA) (C), or ASA/ARC (D), as indicated for 10 min. Platelets were subsequently incubated in the presence of vehicle control or primer, i.e. 100 nm IGF-1 (Ai–Ei), 50 ng mL^−1^ TPO (Aii–Eii), and 5 μm epinephrine (Aiii–Eiii), respectively, for 5 min before stimulation with 2 μm SFLLRN, and aggregation was recorded for 5 min. Representative aggregation traces (A–D) and quantified area under the curve analysis (E) are shown. Data are mean ± standard error of the mean, *n* = 6–8. Statistical analysis: two-way anova was used in conjunction with a Bonferroni *post hoc* test; *P < 0.05, **P < 0.01, ***P < 0.001.

IGF-1 27, TPO 45,46 and epinephrine 29,47 are known to significantly enhance platelet functional responses to physiologic stimuli. In agreement with previous studies, we found that IGF-1, TPO and epinephrine dose-dependently increased SFLLRN-mediated platelet aggregation (Fig. S1). As IGF-1, TPO and epinephrine levels are elevated in various disease states 22,34,35,48–50 and in patients who present with CHD 31–34,36,37, we wanted to evaluate their potential contribution to antiplatelet drug resistance. Platelets were pretreated with ASA, ARC or ASA/ARC in the presence or absence of primers (Fig.2A–E). IGF-1, TPO and epinephrine were unable to activate washed platelets by themselves (Fig. S2), but rescued the inhibitory effect of ASA and/or ARC treatment on SFLLRN-mediated platelet aggregation, as demonstrated by the significant increases in the area under the aggregation curves (Fig.2E). Inhibition of platelet function by ASA was completely rescued by all primers, whereas partial rescue by IGF-1 and TPO was observed in ARC-treated and ASA/ARC-treated platelets. Epinephrine completely rescued ARC-treated platelet function, and significantly rescued the effects of ASA/ARC treatment for both high (Fig.2Eiii) and subthreshold concentrations of SFLLRN (Fig. S3). These results demonstrate the ability of IGF-1, TPO and epinephrine to rescue platelet function in the presence of the antiplatelet compounds ASA, ARC, and ASA/ARC.

### IGF-1, TPO and epinephrine rescue GPVI-mediated platelet function in the presence of antiplatelet compounds

**Figure 3:**
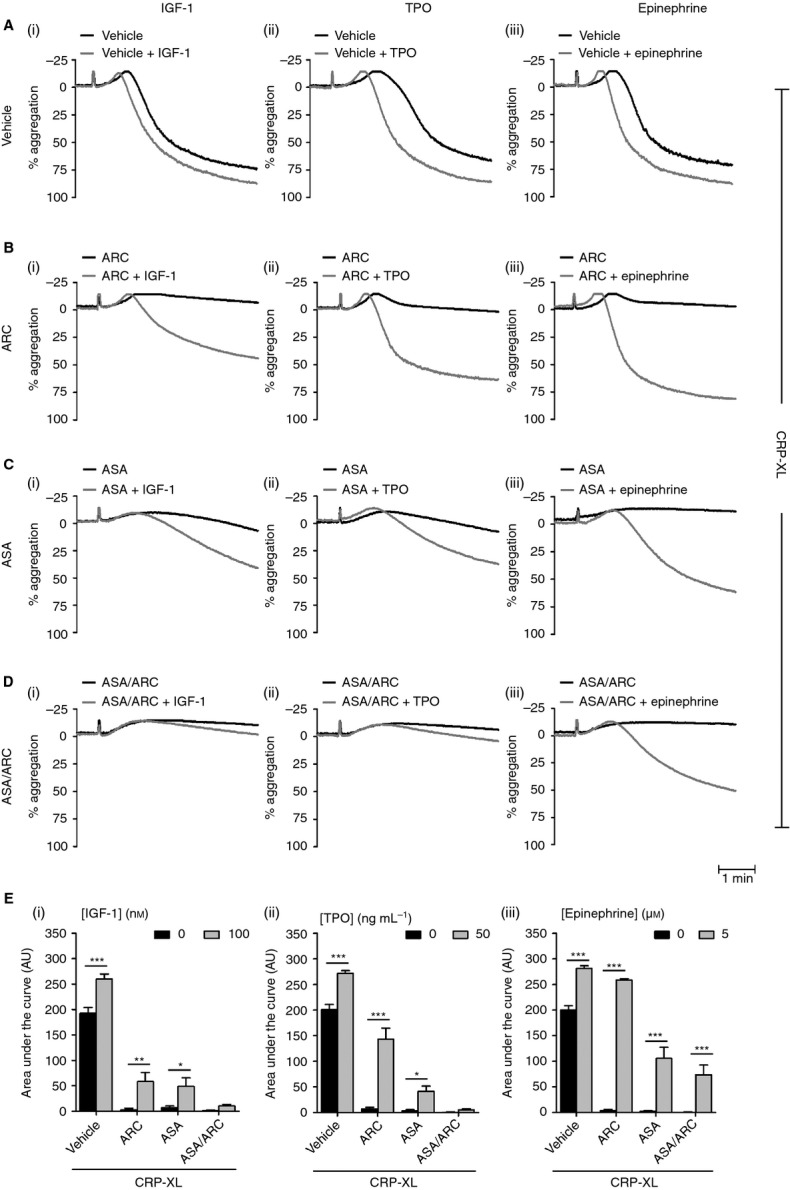
Insulin-like growth factor-1 (IGF-1), thrombopoietin (TPO) and epinephrine rescue glycoprotein VI-mediated platelet function in the presence of antiplatelet compounds. Washed platelets (2 × 10^8^ mL^−1^) were pretreated with vehicle control (HEPES–Tyrode buffer (A) or inhibitors, i.e. 1 μm AR-C66096 (ARC) (B), 30 μm acetylsalicylic acid (ASA) (C) or ASA/ARC (D), as indicated for 10 min. Platelets were subsequently incubated in the presence of vehicle control or primer, i.e. 100 nm IGF-1 (Ai–Ei), 50 ng mL^−1^ TPO (Aii–Eii), and 5 μm epinephrine (Aiii–Eiii), respectively, for 5 min. Platelets were then stimulated with crosslinked collagen-related peptide (0.4–2 μg mL^−1^) to induce an approximately 80% aggregation response, and aggregation was recorded for 5 min. Representative aggregation traces (A–D) and quantified area under the curve analysis (E) are shown. Data are mean ± standard error of the mean, *n* = 6–8. Statistical analysis: two-way anova was used in conjunction with a Bonferroni *post hoc* test; *P < 0.05, **P < 0.01, ***P < 0.001.

Next, we addressed whether primer-mediated resistance to antiplatelet compounds was a PAR-1-specific event. Therefore, we replicated the aggregation studies with an agonist that targets GPVI, i.e. CRP-XL, and pretreated platelets with ASA, ARC or ASA/ARC before spiking with primer (Fig.3A–E). We found that ASA, ARC and ASA/ARC significantly inhibited CRP-XL-mediated platelet aggregation. Similarly to SFLLRN-mediated aggregation, pretreatment with IGF-1, TPO or epinephrine significantly reduced the inhibitory effects of individual treatments with the antiplatelet compounds (Fig.3B–C). Interestingly, epinephrine was the only primer that was able to significantly rescue the inhibitory effect of ASA/ARC treatment (increase from 0.6% ± 0.47% to 73.4% ± 19.3%; area under the curve analysis) (Fig.3Aiii–Eiii). These results demonstrate the ability of primers to rescue the inhibitory effects of individual antiplatelet compounds on CRP-XL-induced GPVI signaling.

### The role of TxA_2_ formation in primer-mediated rescue of the effect of antiplatelet drugs

**Figure 4:**
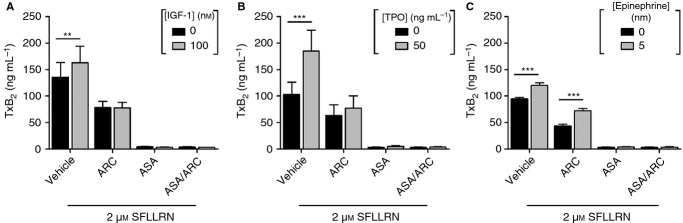
The role of thromboxane A_2_ (TxA_2_) formation in primer-mediated rescue of the effect of antiplatelet drugs. (A) Insulin-like growth factor-1 (IGF-1). (B) Thrombopoietin (TPO). (C) Epinephrine. TxA_2_ production was assessed with a thromboxane B_2_ (TxB_2_) ELISA with releasates generated from the samples shown in Fig.2A–D). Data are presented as mean ± standard error of the mean, *n* = 3–4. Statistical analysis: two-way anova was used in conjunction with a Bonferroni *post hoc* test; **P < 0.01, ***P < 0.001. ARC, AR-C66096; ASA, acetylsalicylic acid.

Antagonism of platelet P2Y_12_ receptors can inhibit platelet activation by inhibiting TxA_2_ production and dampening platelet responses following TxA_2_ receptor activation 51–53. Similarly, ASA blocks COX-1 activity and subsequent generation of TxA_2_ 9. Given the important role of TxA_2_ in platelet activation, we were interested in investigating whether: (i) primers can increase PAR-1-mediated TxA_2_ formation; and (ii) whether this contributes to primer-mediated rescue of PAR-1-mediated platelet function, in particular in the presence of ARC. As expected, ASA and ASA/ARC blocked TxA_2_ production under all conditions. Interestingly, we found that IGF-1, TPO and epinephrine significantly elevated TxA_2_ production in vehicle-treated platelets (Fig.4A–C). However, ARC treatment blocked IGF-1-mediated and TPO-mediated enhancement of TxA_2_ production. In contrast, epinephrine was able to enhance TxA_2_ production in the presence of ARC.

### PI3K plays a critical role in primer-mediated resistance to dual antiplatelet therapy

**Figure 5:**
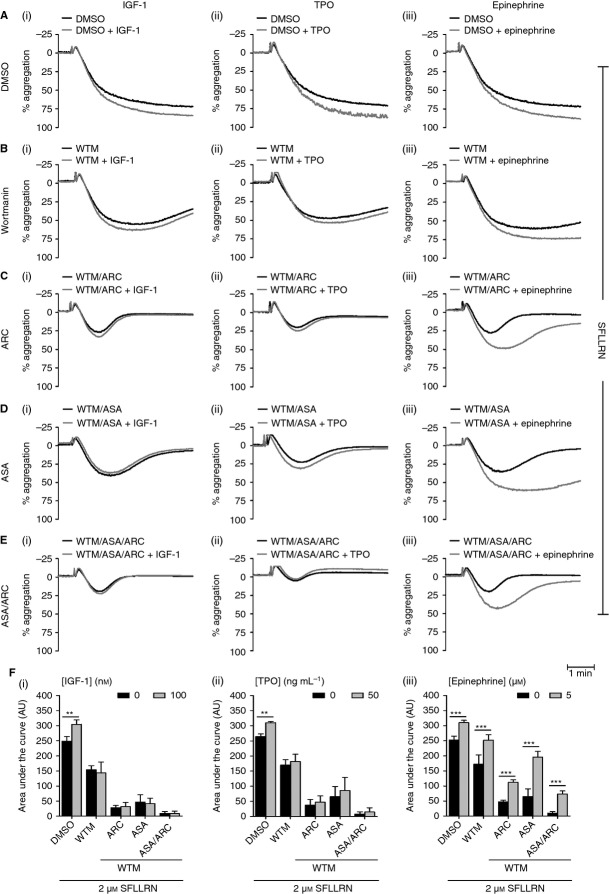
Phosphoinositide 3-kinase plays a critical role in primer-mediated resistance to dual antiplatelet therapy. Washed platelets (2 × 10^8^ mL^−1^) were pretreated with vehicle control (dimethylsulfoxide [DMSO]) (A) or inhibitors, i.e. 100 nm wortmannin (WTM) (B), 1 μm AR-C66096 (ARC) (C), 30 μm acetylsalicylic acid (ASA) (D), or ASA/ARC (E), as indicated for 10 min. Platelets were subsequently incubated in the presence of vehicle control or primer, i.e. 100 nm insulin-like growth factor-1 (IGF-1) (Ai–Ei), 50 ng mL^−1^ thrombopoietin (TPO) (Aii–Eii), and 5 μm epinephrine (Aiii–Eiii), respectively, for 5 min before stimulation with 2 μm SFLLRN, and aggregation was recorded for 5 min. Representative aggregation traces (A–E) and quantified area under the curve analysis (F) are shown. Data are mean ± standard error of the mean, *n* = 3. Statistical analysis: two-way anova was used in conjunction with a Bonferroni *post hoc* test; **P < 0.01, ***P < 0.001.

Various platelet primers have been shown to enhance platelet function via a PI3K-dependent signaling mechanism 26–29,54,55. To investigate whether primer-mediated resistance in SFLLRN-stimulated platelets was mediated by PI3K, we treated platelets with ASA, ARC and/or ASA/ARC in the presence of the pan-PI3K inhibitors wortmannin (Fig.5) and LY294002 (Fig. S4). The results indicated that IGF-1-mediated and TPO-mediated resistance are driven primarily by PI3K, as the addition of wortmannin or LY294002 ablated primer-mediated enhancements in the presence of the antiplatelet compounds (Fig.5Fi,ii; Fig. S4Di,ii)). Interestingly, epinephrine was still able to significantly enhance platelet aggregation in the presence of wortmannin and LY294002 when platelets were treated with various antiplatelet combinations (Fig.5Aiii–Diii; Fig. S4Diii). However, the functional enhancements achieved were more reversible in the presence of the PI3K inhibitors, particularly in the presence of wortmannin, as reflected by a reduction in the rescue effect of epinephrine on ASA/ARC-treated platelets (compare Fig.2Eiii and Fig.5Fiii for area under the curve analysis). Wortmannin reduced aggregation amplitude (*t* = 5 min) in the presence of epinephrine, from 52% ± 3.7% to 8% ± 0.75% (*n* = 3–8). These results demonstrate that PI3K contributes to the later stages of epinephrine-mediated rescue of platelet aggregation; however, epinephrine-mediated resistance appears to be largely PI3K-independent.

### *Ex vivo* thrombus formation on collagen is reduced by dual antiplatelet therapy; a process that is rescued by IGF-1 and epinephrine

**Figure 6:**
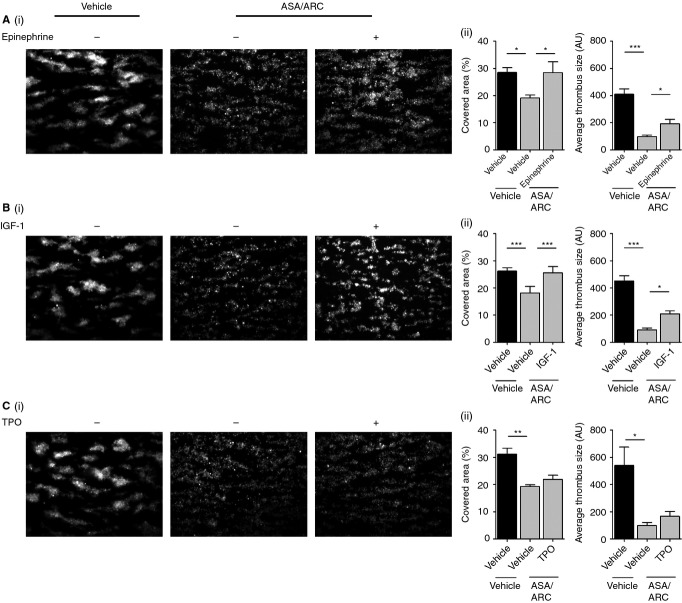
*Ex vivo* thrombus formation on collagen is reduced by dual antiplatelet therapy, a process that is rescued by insulin-like growth factor-1 (IGF-1) and epinephrine. Fluorescently labeled (dihexyloxacarbocyanine iodide [DiOC_6_]) whole blood anticoagulated with 2 U mL^−1^ heparin and 40 μm d-phenylalanylprolyl-arginyl chloromethyl ketone was pretreated with vehicle control or 1 μm AR-C66096 (ARC) and 30 μm acetylsalicylic acid (ASA) in combination for 10 min. Blood samples were subsequently preincubated with vehicle control or primer, i.e. 100 nm epinephrine (A), 100 nm IGF-1 (B), and 50 ng mL^−1^ thrombopoietin (TPO) (C), as indicated for 5 min. Samples were then perfused at an arterial shear rate of 1000 s^−1^ over fibrillar collagen (50 μg mL^−1^) for 5 min. Samples were washed with HEPES–Tyrode buffer for 2 min to remove non-adherent cells. Representative fluorescent images are shown, along with quantitative analysis of surface area covered (%) with thrombi and the average thrombus size (AU) Data represent the average results taken from ≥ 15 random microscopic fields per experiment (*n* = 4–5). Statistical analysis: one-way anova was used in conjunction with a Bonferroni *post hoc* test; *P < 0.05, **P < 0.01, ***P < 0.001.

A recent study has demonstrated that blood from patients receiving dual antiplatelet therapy with P2Y_12_ inhibitors and ASA have a decreased rate of thrombus formation over collagen 56. In agreement with this, we found that exogenous addition of ASA and ARC resulted in a significant reduction in collagen-mediated thrombus formation, as demonstrated by a reduction in the area covered by platelets and the average thrombus size as compared with vehicle control (Fig.6A–C). Platelets pretreated with the antiplatelet compounds also appeared to form thrombi with a loosely packed platelet morphology, similarly to previous findings 57. Interestingly, we found that pretreatment of blood with epinephrine or IGF-1, but not with TPO, reversed the inhibitory effects of the dual antiplatelet compounds, as demonstrated by the complete rescue of the area covered with thrombi (Fig.6A–C). Epinephrine and IGF-1 treatment also increased the average thrombus size, although not to the level of the vehicle control (Fig.6A–C). These results demonstrate that epinephrine and IGF-1 are able to reverse the inhibitory effects of dual antiplatelet treatment and affect the morphology of thrombi.

### PI3K plays a critical role in IGF-1-mediated rescue of *ex vivo* thrombus formation following inhibition with ASA/ARC treatment

**Figure 7:**
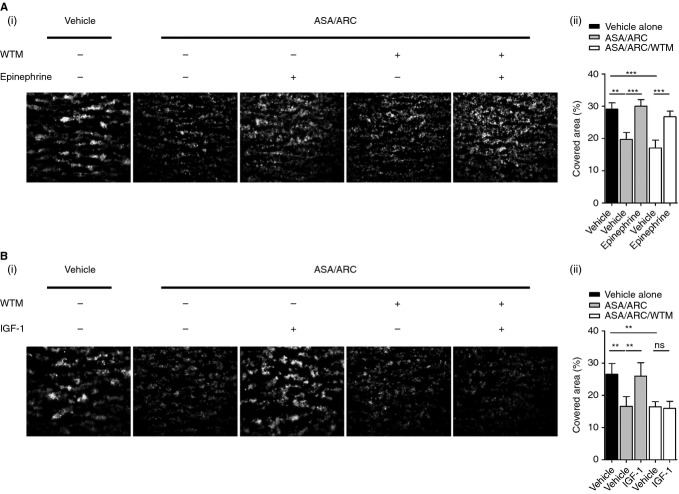
Phosphoinositide 3-kinase plays a critical role in insulin-like growth factor-1 (IGF-1)-mediated rescue of *ex vivo* thrombus formation following inhibition with acetylsalicylic acid (ASA)/AR-C66096 (ARC) treatment. Fluorescently labeled (dihexyloxacarbocyanine iodide) whole blood anticoagulated with 2 U mL^−1^ heparin and 40 μm d-phenylalanylprolyl-arginyl chloromethyl ketone was pretreated with vehicle control or 1 μm ARC and 30 μm ASA in combination in the presence or absence of 100 nm wortmannin (WTM) for a total of 10 min. Blood samples were subsequently preincubated with vehicle control or primer, i.e. 5 μm epinephrine (A) or 100 nm IGF-1 (B), as indicated for a total of 5 min. Samples were then perfused at an arterial shear rate of 1000 s^−1^ over fibrillar collagen (50 μg mL^−1^) for 5 min. Samples were washed with HEPES–Tyrode buffer for 2 min to remove non-adherent cells. Representative fluorescent images are shown, along with quantitative analysis of surface area covered (%) with thrombi. Data represent the average results taken from ≥ 15 random microscopic fields per experiment (*n* = 3–5). Statistical analysis: one-way anova was used in conjunction with a Bonferroni *post hoc* test; **P < 0.01, ***P < 0.001.

To gain some mechanistic insights into the resistance induced by IGF-1 and epinephrine on thrombus formation in whole blood treated with ASA/ARC, we treated samples with the PI3K inhibitor wortmannin (Fig.7). The results demonstrated that epinephrine-mediated resistance to ASA/ARC was largely independent of PI3K activity, as epinephrine was still able to enhance *ex vivo* thrombus formation in the presence of wortmannin (Fig.7A). In contrast, IGF-1-mediated resistance to ASA/ARC was PI3K-dependent (Fig.7B).

### Synergistic effects of IGF-1 and epinephrine on primer-mediated resistance to dual antiplatelet therapy during *ex vivo* thrombus formation

**Figure 8:**
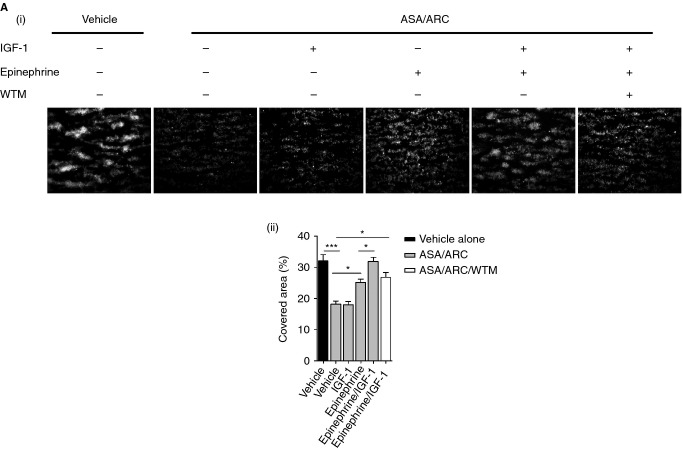
Synergistic effects of insulin-like growth factor-1 (IGF-1) and epinephrine on primer-mediated resistance to dual antiplatelet therapy during *ex vivo* thrombus formation. Fluorescently labeled (dihexyloxacarbocyanine iodide) whole blood anticoagulated with 2 U mL^−1^ heparin and 40 μm d-phenylalanylprolyl-arginyl chloromethyl ketone was pretreated with vehicle control or 1 μm AR-C66096 (ARC) and 30 μm acetylsalicylic acid (ASA) in combination in the presence or absence of 100 nm wortmannin (WTM) for a total of 10 min. Blood samples were subsequently preincubated with vehicle control or primer, i.e. 5 nm IGF-1, 20 nm epinephrine, or a combination of both, for a total of 5 min. Samples were then perfused at an arterial shear rate of 1000 s^−1^ over fibrillar collagen (50 μg mL^−1^) for 5 min. Samples were washed with HEPES–Tyrode buffer for 2 min to remove non-adherent cells. Representative fluorescent images are shown, along with quantitative analysis of surface area covered (%) with thrombi. Data represent the average results taken from ≥ 15 random microscopic fields per experiment (*n* = 3–5). Statistical analysis: one-way anova was used in conjunction with a Bonferroni *post hoc* test; *P < 0.05, **P < 0.01, ***P < 0.001.

It is likely that patients who are susceptible to cardiovascular complications will present with elevations in multiple combinations of circulating primers *in vivo*. We treated whole blood with more physiologic concentrations of IGF-1 (5 nm) and epinephrine (20 nm), alone or in combination, to assess the effects of combined primer treatments on antiplatelet resistance. We found that 5 nm IGF-1 alone was unable to enhance thrombus formation in the presence of ASA/ARC, whereas 20 nm epinephrine was still able to significantly increase the area covered with thrombi (Fig.8). Interestingly, combined treatment with 5 nm IGF-1 and 20 nm epinephrine had synergistic rescuing effects, with full recovery of the area covered with thrombi in the presence of ASA/ARC. Furthermore, the morphology of the thrombi formed was similar to that of the thrombi achieved with the vehicle control. The synergistic effect of IGF-1 treatment in combination with epinephrine was blocked by wortmannin, with the area covered by thrombi being comparable to the rescue achieved with epinephrine alone. This further confirms the important role of PI3K in IGF-1-mediated resistance to antiplatelet therapy.

## Discussion

Aspirin and P2Y_12_ antagonists are commonly administered to patients at risk for thrombosis. Certain patient populations show resistance to these antiplatelet compounds, and are at risk of developing subsequent cardiovascular complications 58. In this study, we determined the role of platelet primers in resistance to antiplatelet therapy. We found that the primers IGF-1, TPO, and epinephrine, which are known to be elevated in patients with or at risk of ACS, were able to overcome the inhibitory effects of antiplatelet drugs on platelet functional responses 31–34,36,37. Furthermore, we have demonstrated that IGF-1-mediated and TPO-mediated resistance to antiplatelet drugs is PI3K-dependent, as pan-PI3K inhibitors blocked this resistance. Interestingly, PI3K inhibition did not block epinephrine-mediated resistance, revealing a PI3K-independent mechanism of resistance.

One of the proposed mechanisms by which antiplatelet resistance develops is via activation of alternative signaling pathways that are ADP-independent and/or TxA_2_-independent 59. Interestingly, we found that platelet primers were able to enhance functional responses in the presence of ADP and TxA_2_ inhibitors. This demonstrates that there are ADP-independent and TxA_2_-independent mechanisms driving primer-mediated resistance to antiplatelet compounds. Indeed, several studies found priming effects of IGF-1, TPO and epinephrine on platelet function under conditions where the ADP receptor P2Y_12_ was blocked, confirming that ADP release is not essential in this process 27,28,60,61. In contrast, other studies have shown that the effect of TPO on platelet function is, at least partially, mediated by an increase in TxA_2_ production 62. Here, we have demonstrated that IGF-1, TPO and epinephrine all increase SFLLRN-mediated TxA_2_ production. However, it is unlikely that this increase in TxA_2_ formation contributes to primer-mediated resistance to antiplatelet compounds, because: (i) IGF-1-mediated and TPO-mediated increases in TxA_2_ formation were blocked by ARC; and (ii) although epinephrine-mediated increases in TxA_2_ formation were still present in ARC-treated platelets, the finding that ASA had little effect on epinephrine-mediated rescue of the inhibitory effect of ARC (Fig.2Eiii) suggests that increased TxA_2_ formation plays a minor role in this context. These findings confirm that primer-mediated resistance to ARC is largely independent of TxA_2_ formation.

We translated our studies into a more physiologic setting to assess the effects of treatment of whole blood with ASA/ARC on *ex vivo* thrombus formation. ASA/ARC treatment significantly reduced the area covered with thrombi and the average thrombus size. Notably, ASA/ARC treatment had a distinct effect on thrombus morphology, whereby individual platelets could be clearly identified. This observation is in agreement with other studies, which have also shown the ability of the P2Y_12_ inhibitor AR-C69931MX (cangrelor) to reduce platelet thrombus height 63. Furthermore, platelets from P2Y_12_-deficient mice that have been treated with aspirin show a similar arrangement of loosely packed thrombi during flow studies 57. As TxA_2_ and ADP play an important paracrine/autocrine role that is involved in stabilizing thrombi 64,65, it may not be surprising that aspirin and P2Y_12_ blockade gives rise to a loosely packed platelet morphology.

Epinephrine and IGF-1 rescued the inhibitory effects of ASA/ARC, significantly increasing not only the area covered with thrombi, but also the thrombus size. Although epinephrine-treated platelets formed larger thrombi, they retained their loosely packed morphology. Conversely, IGF-1 treatment appeared to give rise to thrombi that were more comparable to those obtained with the vehicle control, with the identification of individual platelets being more difficult. TPO was unable to rescue the inhibitory effects of ASA and P2Y_12_ blockade, demonstrating that TPO-mediated potentiation of GPVI platelet function is reliant on TxA_2_ and ADP signaling.

Numerous platelet primers, including matrix metalloproteinase-2, Gas6, ephrin B, IGF-1, TPO, and epinephrine, have been reported to enhance platelet function via a PI3K-dependent signaling mechanism 26–29,54,55. To establish the role of PI3K in primer-mediated resistance to antiplatelet compounds, we treated platelets with pan-PI3K inhibitors, and we observed complete blockade of IGF-1-mediated and TPO-mediated potentiation of SFLLRN-induced aggregation in the presence of ASA/ARC. This observation is consistent with previous studies that demonstrated an important role of PI3K in IGF-1-mediated and TPO-mediated potentiation of platelet function 26–28,61,66, and reveals a critical role of PI3K in IGF-1-mediated and TPO-mediated resistance to ASA and P2Y_12_ antagonism. It is likely that the p110α isoform of PI3K drives IGF-1-mediated resistance to antiplatelet compounds, as previous studies have revealed an important role of this isoform in IGF-1-mediated potentiation of platelet function 26,27,61. Interestingly, PI3K inhibitors were unable to block epinephrine-mediated enhancement of platelet function in the presence of ASA/ARC, revealing a PI3K-independent mechanism for epinephrine-mediated resistance. Epinephrine binds to the α2A-adrenergic receptor, and stimulates activation of Gz signaling pathways, which may substitute for Gi-coupled P2Y_12_ signaling during platelet activation 67. Therefore, epinephrine may compensate for blockade of ADP, resulting in resistance to P2Y_12_ antagonists. It is of note that several studies found a correlation between exaggerated platelet responses to low doses of epinephrine and platelet hyperactivity 68,69, indicating that epinephrine may contribute to platelet hyperactivity. Epinephrine has also been found to potentiate Ca^2+^ release in platelets; therefore, we explored the role that Ca^2+^ may play in epinephrine-mediated resistance to antiplatelet compounds 70. Our results demonstrated that elevations in intracellular Ca^2+^ did not contribute to epinephrine-mediated resistance or to the resistance induced by IGF-1 and TPO (Fig. S5).

IGF-1-mediated enhancement of thrombus formation in the presence of ASA/ARC was blocked by wortmannin. However, epinephrine was still able to rescue the inhibitory effects of the antiplatelet compounds in the presence of wortmannin. This reinforces the role of PI3K in IGF-1-mediated resistance, and further demonstrates the PI3K-independent mechanisms driving epinephrine-mediated resistance. Interestingly, combined treatment with more physiologic levels of IGF-1 (5 nm) and epinephrine (20 nm) gave rise to synergistic rescuing effects in the presence of ASA/ARC. Although IGF-1 (5 nm) was unable to rescue the inhibitory effects of ASA/ARC alone, it was able to enhance the rescuing effects of epinephrine (20 nm) and return the morphology of the thrombi to that seen with the vehicle. This was a particularly interesting observation, as it is likely that patients who present with ACS or who are susceptible to ACS will have elevations in multiple circulating primers. Primers may act independently to make patients resistant to antiplatelet therapy; however, it is more likely that combinations of primers have additive or synergistic effects that put patients at higher risk for thrombotic vascular events.

In conclusion, we have demonstrated the ability of circulating primers to overcome the inhibitory effects of ASA and/or ARC. Changes in plasma levels of primers therefore predispose patients to antiplatelet resistance and thrombotic events. We have shown that there are PI3K-dependent mechanisms driving IGF-1-mediated and TPO-mediated resistance, whereas PI3K-independent mechanisms drive epinephrine-mediated resistance. The PI3K pathway and the α2A-adrenergic receptor may be promising drug targets to combat the insufficient inhibition induced by current antiplatelet therapies.

## Addendum

T. A. Blair designed and performed experiments, analyzed the results, and wrote the manuscript. S. F. Moore designed and performed initial experiments, contributed to discussion, and edited the manuscript. I. Hers conceived the experiments, supervised the project, and wrote the manuscript.

## Supporting Information

**Fig. S1.** IGF-1, TPO and epinephrine potentiate platelet aggregation in a dose-dependent manner.

**Fig. S2.** IGF-1, TPO and epinephrineA do not act as agonists in the washed platelet system.

**Fig. S3.** Epinephrine and combined primer treatments rescue PAR-1-mediated platelet aggregation induced by subthreshold SFLLRN in the presence of antiplatelet compounds.

**Fig. S4.** The PI3K inhibitor LY294002 reveals a critical role of PI3K in IGF-1-mediated and TPO-mediated resistance to dual antiplatelet therapy.

**Fig. S5.** Primer-mediated resistance to antiplatelet drugs is not driven by Ca^2+^ signaling.
