# Supplementary figures and images for: Circulating primers enhance platelet function and induce resistance to antiplatelet therapy
Source: J Thromb Haemost. 2015 Jun 25;13(8):1479–93. doi: 10.1111/jth.13022 (PMC4599128; doi:10.1111/jth.13022)

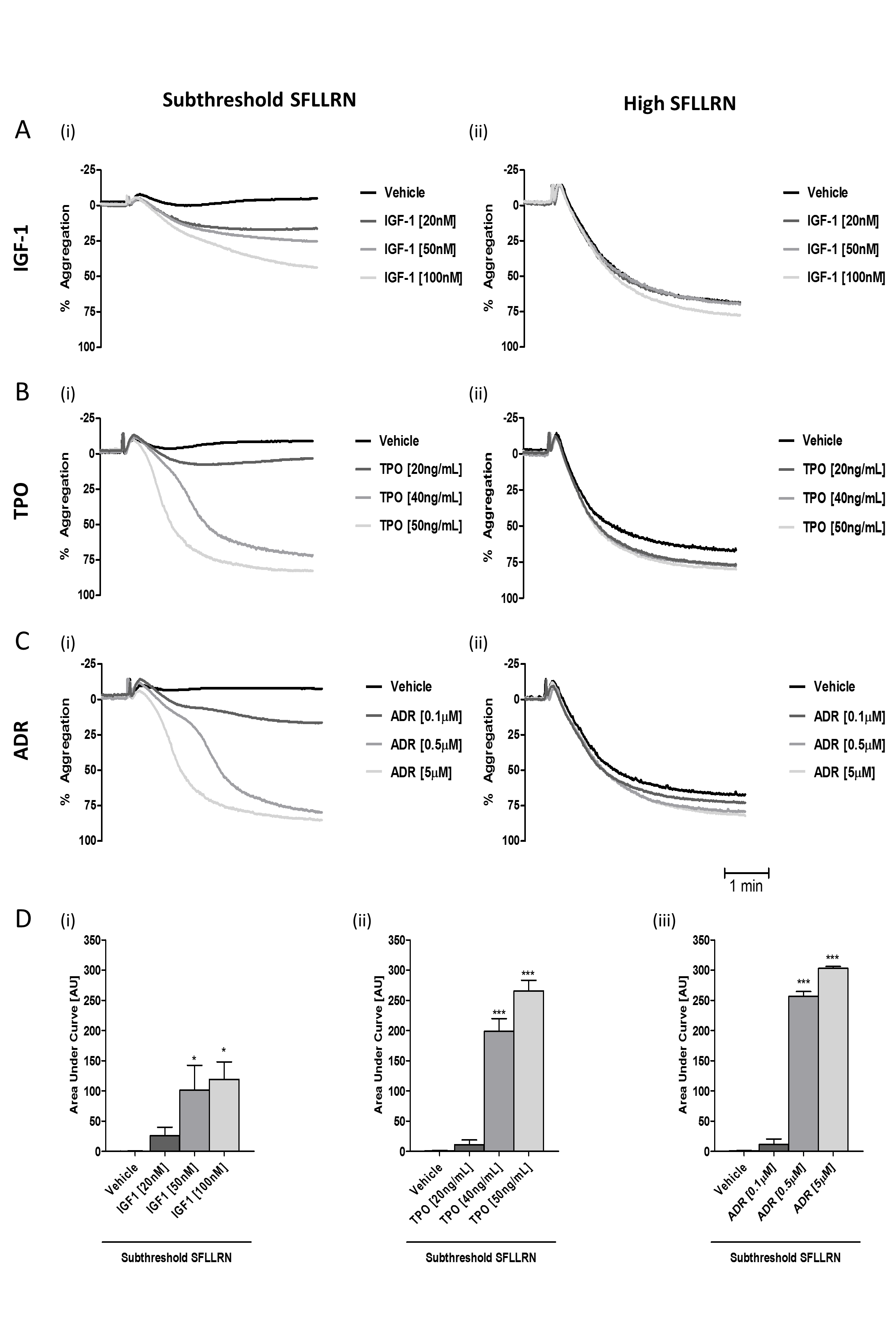

Supplement: Supplementary file 1 — Fig. S1. IGF-1, TPO and epinephrine potentiate platelet aggregation in a dose-dependent manner. [file jth0013-1479-sd1.tif]

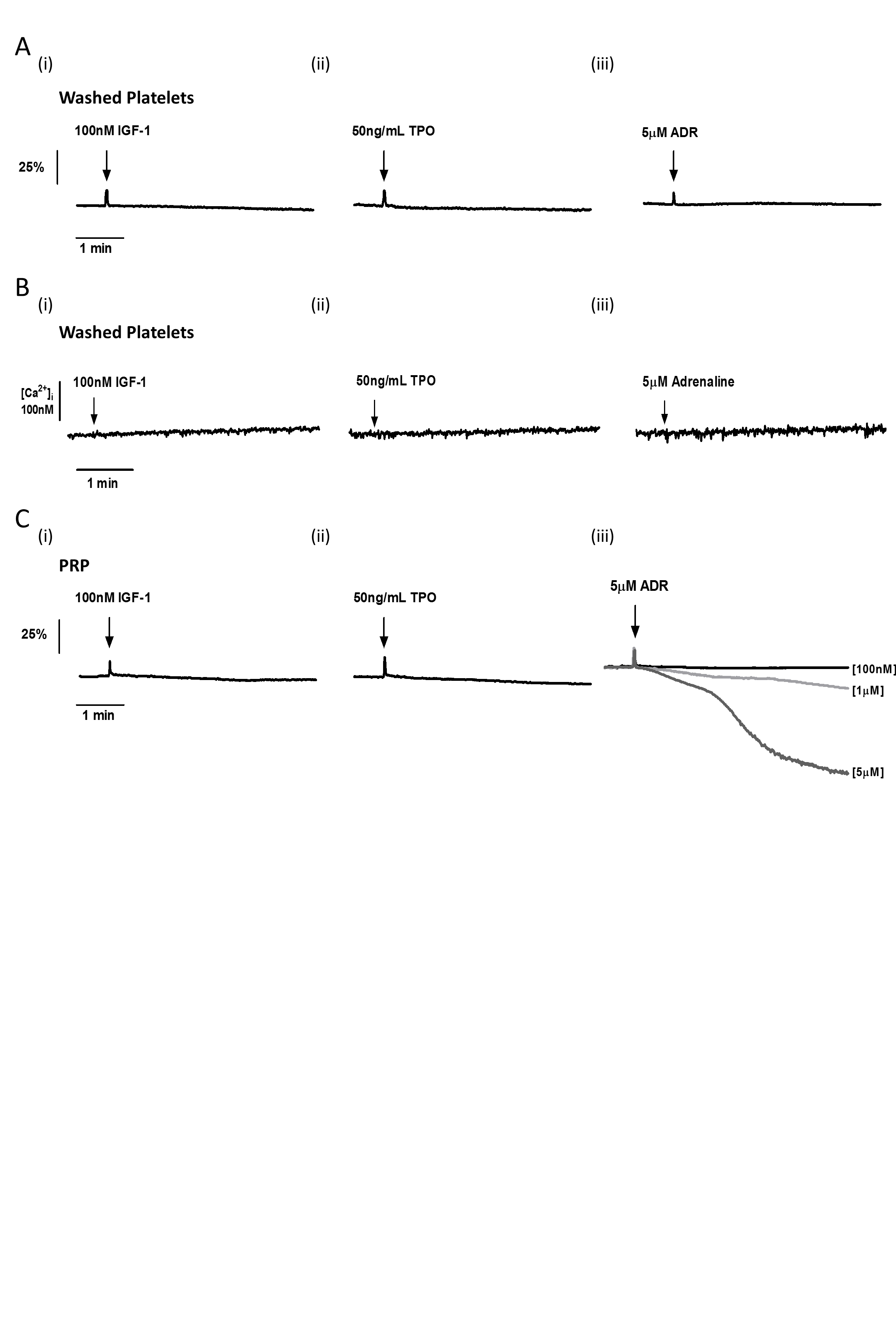

Supplement: Supplementary file 2 — Fig. S2. IGF-1, TPO and epinephrineA do not act as agonists in the washed platelet system. [file jth0013-1479-sd2.tif]

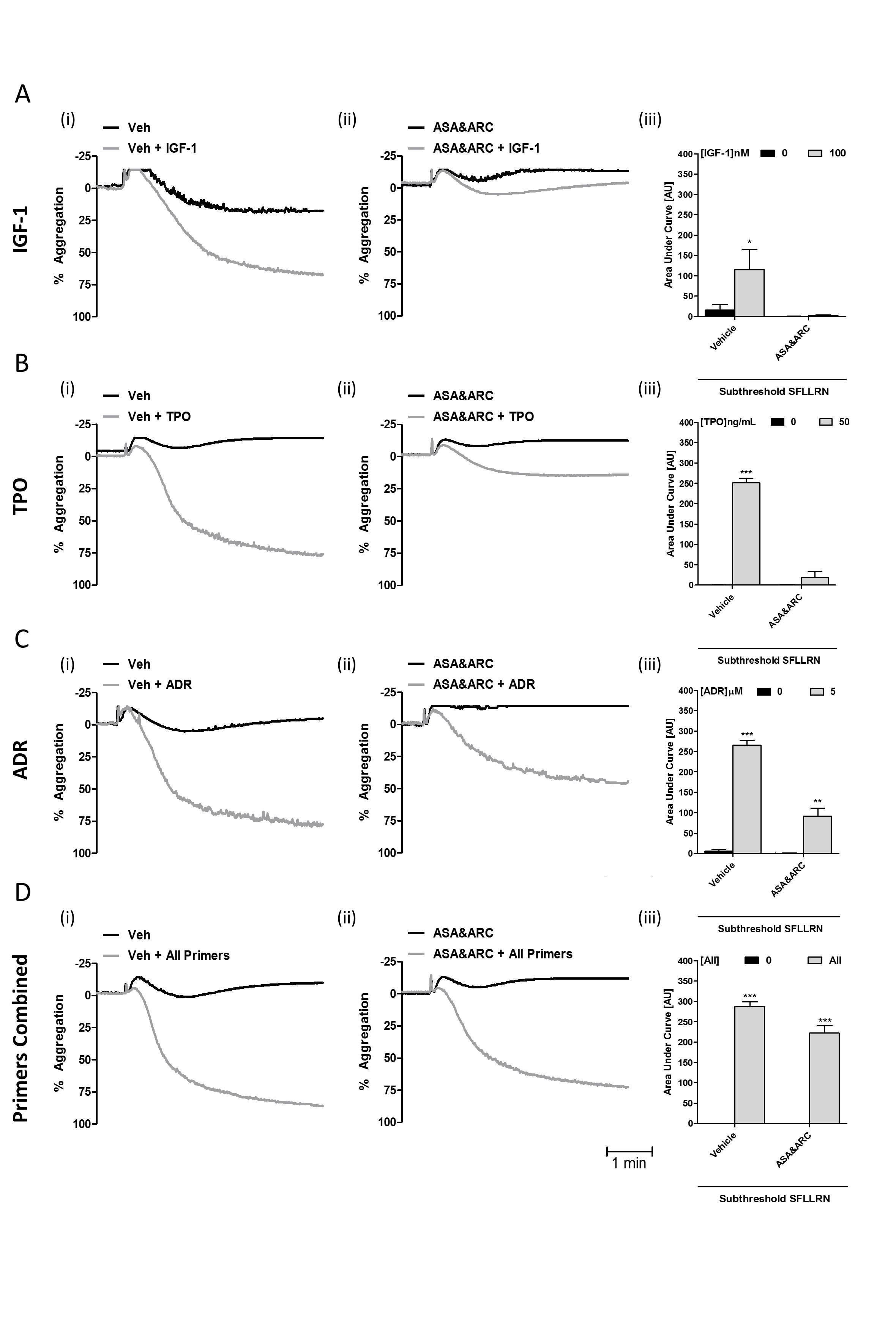

Supplement: Supplementary file 3 — Fig. S3. Epinephrine and combined primer treatments rescue PAR-1-mediated platelet aggregation induced by subthreshold SFLLRN in the presence of antiplatelet compounds. [file jth0013-1479-sd3.tif]

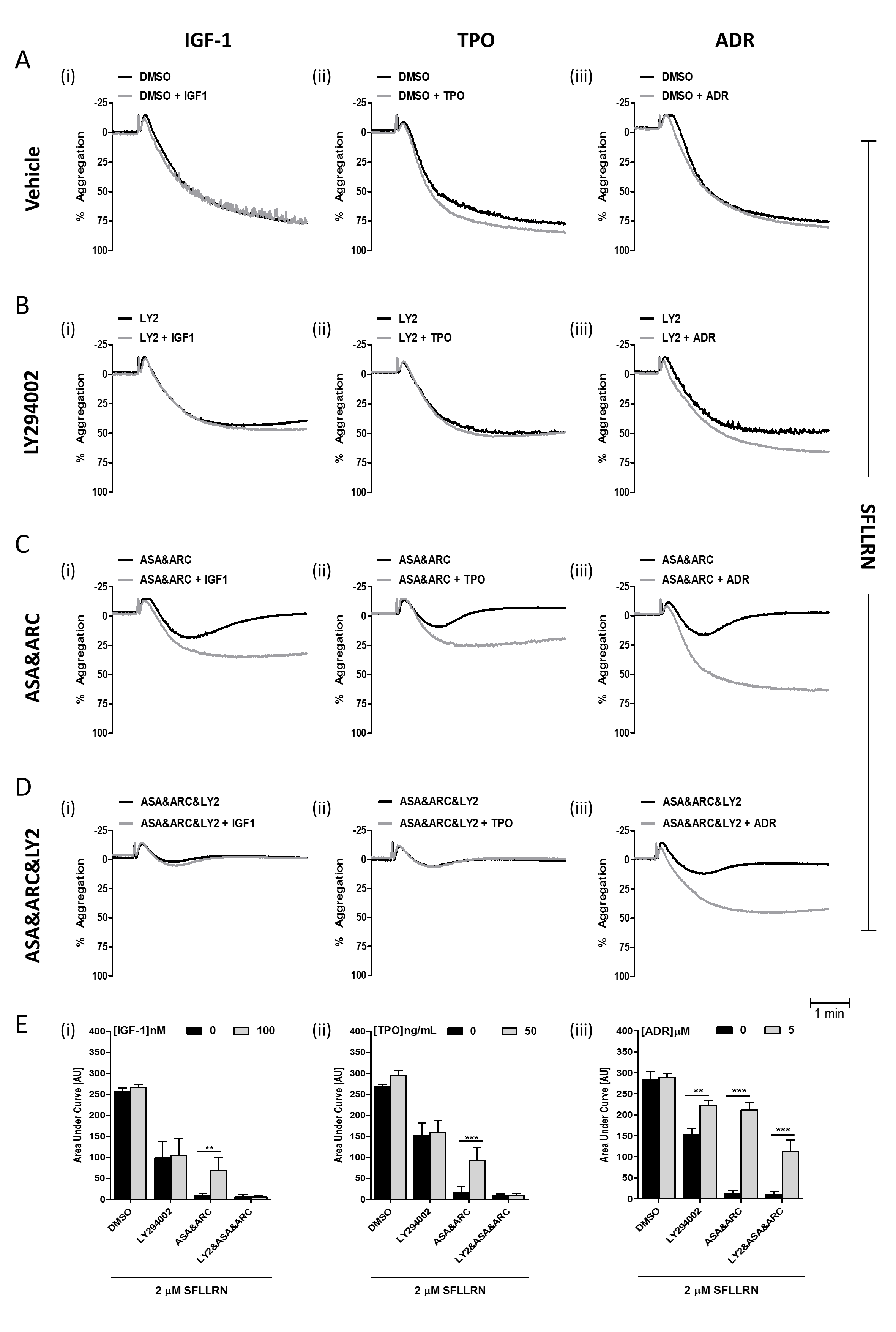

Supplement: Supplementary file 4 — Fig. S4. The PI3K inhibitor LY294002 reveals a critical role of PI3K in IGF-1-mediated and TPO-mediated resistance to dual antiplatelet therapy. [file jth0013-1479-sd4.tif]

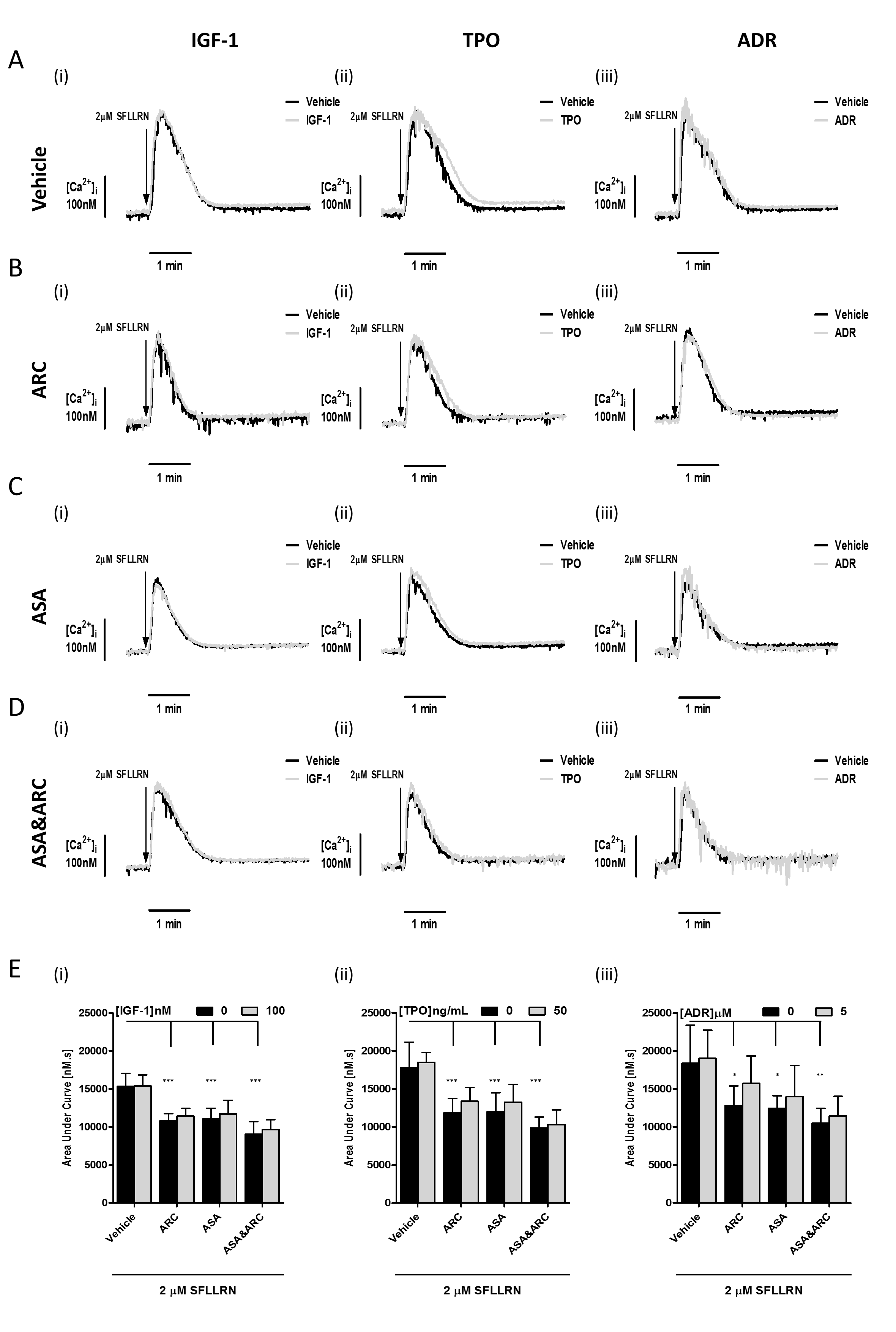

Supplement: Supplementary file 5 — Fig. S5. Primer-mediated resistance to antiplatelet drugs is not driven by Ca2+ signaling. [file jth0013-1479-sd5.tif]
